# Limited predictive value of pre-surgical level of functioning for functioning at 3 and 12 months after TKA

**DOI:** 10.1007/s00167-018-5288-5

**Published:** 2018-11-28

**Authors:** Danielle D. P. Berghmans, Antoine F. Lenssen, Pieter J. Emans, Lodewijk W. van Rhijn, Rob A. de Bie

**Affiliations:** 1 Department of Physical Therapy, Maastricht University Medical Center, Maastricht, The Netherlands; 2 MUMC+, 5800, 6202 AZ Maastricht, PO The Netherlands; 3 Department of Orthopedics, Maastricht University Medical Center, Maastricht, The Netherlands; 4 Department of Epidemiology, Maastricht University, Maastricht, The Netherlands; 5 Maastricht University, P.O. Box 616, 6200 MD Maastricht, The Netherlands; 6 CAPHRI School for Public Health and Primary Care, Maastricht University, Maastricht, The Netherlands

**Keywords:** Osteoarthritis, Arthroplasty, Knee replacement, TKA

## Abstract

**Purpose:**

A total knee arthroplasty (TKA) is a cost-effective option to relieve pain and improve knee function in patients suffering from osteoarthritis. However, results differ among patients. The predictive value of pre-surgically assessed factors on the level of functioning after 3 and 12 months was investigated in this study.

**Methods:**

This study used an inception cohort design and a follow-up of 12 months. One hundred and fifty patients who were to receive a TKA were assessed pre-surgically with an International Classification of Functioning, Disability and Health (ICF) core assessment set: Knee Society Score (KSS), Western Ontario and McMaster Universities Osteoarthritis Index (WOMAC), Short-Form 12 (SF12), Patient-Specific Function Scale (PSFS), range of motion (ROM), quadriceps and hamstring strength and gait parameters. The main outcome measure was WOMAC-Function at 3 and 12 months after surgery.

**Results:**

Pre-surgical physical and mental health on the SF12 and functioning and stiffness on the WOMAC explained 23% of the variance in the level of functioning 3 months after surgery. Pre-surgical knee function measured with the KSS-Knee, and functioning as assessed by WOMAC-Function explained 16% of the variance of the level of functioning 12 months after surgery.

**Conclusions:**

The results of this study show that better functioning before surgery, less knee stiffness and a better physical and mental health to some extent predict better functioning 3 months after surgery. This effect is less evident at 12 months. This study is clinically relevant since it provides benchmark data for health care providers who want to compare their individual patients.

**Level of evidence:**

II.

## Introduction

Several studies have investigated the influence of pre-surgical predictors on functional status post surgery. They found that scores on post-surgical health status questionnaires concerning level of functioning or quality of life are influenced by demographic factors [1, 7, 12, 21] (age, sex, body composition), psychosocial factors, [1, 7, 12, 15, 21] medical factors (e.g. previous surgeries, complications, comorbidities), [7, 11, 15, 21] baseline physical functioning, [1, 6, 11, 12, 15, 18, 21] use of walking devices, [11] walking distance [11] and pre-surgical pain [6, 15, 18].

However, existing evidence remains suboptimal, which is partly due to differences in study designs, specifically differences in prediction time periods [1, 3, 11, 12, 21], predictors [6, 7, 11, 12, 15, 18, 21] and outcome variables [1, 12], as well as small study populations [3, 21]. Low quality evidence was also reported in a systematic review by Harmelink and Zeegers et al. [8] of prognostic factors for pain, functioning and quality of life 1 year after TKA surgery. As far as we know, no study has combined self-perceived questionnaires with a wide range of physical tests in a large population. Furthermore, no other study has objectively measured gait parameters in an extensive group of patients and used it in a prediction model [19]. This information is clinically relevant to determine the overall status of a patient. Recently there has been a great deal of interest regarding the effect of improving pre-surgical status on the immediate post-surgical recovery [23]. In the present study, the relationship between pre-surgical functional parameters and functional ability in the longer term was examined. Large datasets regarding the pre- and post-surgical functional status of TKA patients are lacking, which makes our study unique. In addition, the large study sample enabled us to investigate the relationship between multiple parameters and the post-surgical functional status. Our hypothesis was that taking account of pre-surgical functional parameters and self-report questionnaires assessing the ‘function’ and ‘disability’ domains of the International Classification of Functioning, Disability and Health model (ICF) would make the prediction models more complete. This information could inform clinicians and patients in the pre-surgical process.

A prognostic cohort study was conducted to determine the predictive value of factors measured before surgery for the level of functioning (measured with the Western Ontario and McMaster Universities Osteoarthritis Index function scale [WOMACF]) 3 and 12 months after a TKA.

The hypothesis was that a combination of pre-surgical functions and demographic variables would predict the post-surgical level.

## Materials and methods

The local medical ethics committee reviewed and approved the study (NL33015.068.10/METC 10-2-083). The rights of subjects were protected under the Declaration of Helsinki.

An inception cohort design was used to recruit all consecutive patients with end-stage osteoarthritis 1 day before surgery. All patients were assessed as described in the “Procedure” section, before surgery. The WOMAC was repeated at 3 and 12 months after surgery, during a personal follow-up contact.

### Patients

One hundred and fifty consecutive patients with osteoarthritis of the knee scheduled for a TKA at the Maastricht University Medical Centre (MUMC+) were informed about the study in writing and verbally at least 1 week before the planned surgery. The day before their surgery, all patients to be included were contacted by the researcher, and written informed consent was obtained. Four patients were lost to follow-up in the first prediction model (up to 3 months), and two additional patients were lost to follow-up in the second model (up to 12 months).

Inclusion criteria were: Dutch-speaking patients aged between 18 and 80 years at the time of surgery, diagnosed with osteoarthritis of the knee for which primary TKA was indicated. Patients were excluded if they underwent a unicondylar knee arthroplasty, had a neurological problem influencing ambulation or had an immobile hip or ankle arthrodesis. In addition, severe comorbidities (including severe psychological comorbidities) were automatically excluded because all patients had to be eligible for surgery.

The study population consisted of 79 women and 71 men. Mean age was 64.7 years (± 7.9). Table 4 (appendix) shows baseline values for all parameters. The number of patients differed somewhat between the measurement instruments, because of inability to test due to unavailability or malfunction of the assessment equipment, or due to patients’ inability (only regarding the quadriceps and hamstrings isometric 30° measurements, in patients whose ROM was limited).

### Surgery

All operations were performed by two orthopaedic surgeons, both with extensive experience with the procedure and prosthesis. All patients received a cemented Scorpio or Scorpio NRG Knee System (Stryker, Kalamazoo, Michigan, USA). After a medial parapatellar approach, a bony referenced, tibia-first technique was used. A cemented patella component was placed in 21 patients and a tourniquet was only used during the cementation period of the prosthesis. A previous study reported no differences in ROM, functioning or quality of life between the two prostheses [17].

### Procedure

After signing informed consent, patients were enrolled in the study. All assessments were performed by the research team at the hospital on the day before surgery. The WOMAC was reassessed after 3 and 12 months. Patients were not shown the answers they had given at baseline or at 3 months.

All factors of the function and disability level of the ICF Model were included. The WOMACF was chosen as a primary outcome measure, as this scale comprises a variety of activities that are important in daily life.

Health insurance is mandatory in the Netherlands, so there were no financial obstacles. Patients who were unable to return to their own homes after surgery had the option of going to a rehabilitation facility.

After surgery, patients received per protocol physical therapy in the hospital phase, aimed at increasing functional independence. After patients had left the hospital, physical therapy was continued in a private practice setting and patients received therapy according to the Dutch guidelines on TKA [13].

### Measurements

In addition to assessing the patients’ demographic characteristics (age, sex, height and weight), the following measurements were performed by the physical therapy team, using a standardized protocol.

#### Health status questionnaires

The *WOMAC* is a self-administered disease-specific health questionnaire designed to measure functional ability of the osteoarthritic hip and knee. The WOMAC provides aggregate scores for each of 3 subscales: joint pain, joint stiffness and function. Together, they form the total WOMAC score. The WOMAC is a responsive instrument that yields reliable and valid measurements in a population of patients with hip and knee osteoarthritis and has been used extensively to evaluate this patient population [11]. The 5-point Likert version of the WOMAC was used in our study (scale 0 to 100 points, 100 = best score). The baseline sub-scores were used as parameters in this study, while the function sub-score of the WOMAC at 3 and 12 months was used as the outcome measure.

The *Patient Specific Function Scale (PSFS)* is a questionnaire to record patients’ perceptions of their disabilities [22]. Patients define their main complaints regarding activities and rate the difficulty of performance on an 11-point numerical rating scale (NRS) [22]. The PSFS is a reliable and responsive measure in this population [2].

The *Knee Society Score* (*KSS*) is a knee-joint specific questionnaire and consists of two parts: a knee score and a function score [9]. The KSS is a valid and responsive measure in a population of patients following TKA [16].

The *Short Form 12* (*SF12*) is a generic multidimensional questionnaire measuring quality of life from patients’ point of view. It is a short version of the SF36 and includes two components (physical and mental health), representing their respective domains. It is a valid, reliable and responsive measure in a general population [24].

#### Physical performance test

*Muscle strength* was assessed with a Biodex® System 3 Pro dynamometer, measuring isometric (30° and 60°, in Nm, 3 repetitions each) and isokinetic peak torques (velocities of 60° and 180°/second, in Nm, 5 and 10 repetitions, respectively) of the quadriceps and hamstrings. The Biodex® is a reliable and valid instrument [4].

*Range of motion* (ROM) was measured with a long-arm goniometer according to Lenssen et al. [14] Extension and flexion were measured in supine position, with hyperextension recorded as positive values. Measuring ROM is reliable at group level in patients after a TKA [14].

The *gait parameters* were measured with the GAITRite® system, a highly valid and reliable tool for measuring gait parameters in patients undergoing a TKA [25]. An electronic walkway is connected to a computer via six pressure-activated sensor pads inserted in a roll-up carpet.

### Statistical analyses

At the start of the study, a sample size calculation was performed. Based on the number of determinants and the pragmatic rule of thumb to include ten cases for each determinant under study, at least 120 cases would be needed to obtain sufficient power (10 × 12 determinants = 120). Since some loss-to-follow-up was expected, the total number needed was 150 subjects.

All data were collected. Missing values were not substituted and drop-outs were not replaced. All analyses were performed with SPSS version 23 [5]. Means and standard deviations were calculated.

Univariate regression analysis was performed including WOMACF and all independent variables. Factors with a *p*-value ≤ 0.10 were entered into the multivariate regression model, using the ENTER method according to Field [5]. Assumptions were checked by residual plots and statistics, and total WOMAC scores were not included as predictors because of possible collinearity. Two prediction models were constructed: one model to predict level of functioning as measured with the WOMACF at 3 months, and another for functioning at 12 months.

The following independent variables measured before surgery were used: (1) demographic variables (age, sex), (2) BMI, (3) PSFS, (4) SF12-Physical, (5) SF12-Mental, (6) quadriceps strength, (7) hamstrings strength, (8) gait parameters, (9) ROM, (10) WOMAC at baseline.

## Results

The pre-surgical values of all parameters are provided in Appendix Table 4, giving means and 95% confidence intervals. The average overall improvement on the dependent variable of this study, the WOMACF, was 39.3% after 3 months and 51.0% after 12 months (Table 1).

**Table 1:** Mean values of the WOMAC subscales at baseline and 3 and 12 months post TKA

	Baseline			3 months			12 months		
	*n*	Mean	95% CI	*n*	Mean	95% CI	*n*	Mean	95% CI
WOMAC-pain	149	10.7	10.0–11.4	146	16.0	15.3–16.7	144	17.6	16.8–18.4
WOMAC-stiffness	149	4.1	3.8–4.4	146	4.9	4.6–5.2	144	5.8	5.5–6.1
WOMAC-function	149	39.0	36.9–41.1	146	54.4	52.2–56.6	144	58.2	56.0–60.4
WOMAC-total	149	54.1	51.4–56.8	146	75.4	72.4–78.4	144	81.7	78.6–84.8

*n* number, *95%CI* 95% confidence interval

The results of the univariate regression analysis including pre-surgical parameters and the WOMACF are presented in Appendix Table 5, which only shows the parameters that were significant and retained for multivariate analysis. As mentioned in the *Methods* section, only parameters with a significance level ≤ 0.10 were included in the multivariate regression analysis. Since multiple parameters were significant for WOMACF at 3 and 12 months, several parameters were included in both models.

The final prediction model for level of functioning after 3 months, obtained from the multivariate regression analysis using the ENTER method, is presented in Table 2. This model had an *R*^2^ of 0.228.

**Table 2:** Prediction model for level of functioning 3 months after TKA

	*B*	Std. error	*p*-value	95% CI LB	95% CI UB
Constant	143.40	58.10	0.01	28.52	258.28
SF12P	0.27	0.16	0.10	− 0.05	0.58
SF12M	0.27	0.10	0.01	0.08	0.47
WOMACS	− 1.16	0.65	0.08	− 2.44	0.13
WOMACF	0.44	0.11	0.00	0.22	0.66

*95% CI LB* 95% confidence interval lower bound, *95% CI UB* 95% confidence interval upper bound

Table 3 shows the final prediction model for functioning level after 12 months, after multivariate regression analysis with significant parameters from the univariate analysis and this final model had an *R*^2^ of 0.163.

**Table 3:** Prediction model for level of functioning 12 months after TKA

	*B*	Std. error	*p*-value	95% CI LB	95% CI UB
(Constant)	57.07	5.91	0.00	45.38	68.76
WOMACF	0.36	0.09	0.00	0.18	0.53
KSSK	0.12	0.07	0.09	-0.02	0.25

*Std. Error* standard Error, *95% CI LB* 95% confidence interval lower bound, *95% CI UB* 95% confidence interval upper bound

## Discussion

The most important finding of this study was that an extensive pre-surgical set of measurements including relevant parameters of the functions and disabilities domains of the ICF model only had a limited predictive value for the level of functioning 3 and 12 months after TKA. The prediction model, which included SF12-Mental, SF12-Physical, WOMACS and WOMACF, explained 22.8% of the variance in the level of functioning at 3 months. This implies that the mental and physical status of the patients before surgery, combined with the degree of knee stiffness and the level of pre-surgical functioning predicted nearly a quarter of the level of functioning 3 months after surgery. This implies that better functioning before surgery, less knee stiffness and a better physical and mental health to some extent predict better functioning in the longer term.

The prediction model for 12 months after TKA surgery only included two predictors: WOMACF and KSSK, and explained 16.3% of the variance. In contrast to the model for 3 months, the patients’ baseline mental status and overall physical status had no predictive value for the outcome after 12 months. Instead, only the pre-surgical level of functioning and the pre-surgical overall knee status predicted a mere sixth of the level of functioning 1 year after surgery. Both predictors are influenced by the timing of surgery; if the osteoarthritis worsens, the knee stiffens and muscle strength deteriorates, so the level of pre-surgical functioning is lower. After the TKA surgery there is then a higher risk that the level of functioning will still be lower after 1 year. Further research into the best timing for surgery would be helpful.

The constant factor in both our models was high, implying that the entire study sample showed good progress in terms of level of functioning. This supports the general concept of TKA as an effective procedure in osteoarthritis of the knee. The influence of the individual factors was small, as has also been reported by Jiang, Sanchez et al. [10] The small percentage of variance explained by both models is in line with what was reported in previous studies regarding the prediction of the level of functioning after a TKA using parameters measured before surgery. This means that a large part of the level of functioning could not be explained by physical predictors, neither in our study nor in those reported in the literature [6, 10, 11, 21, 26]. Therefore, the influence of other factors, not taken into account in this study, such as surgical techniques, complications or comorbidities, could explain another part of the variance and should be investigated in future studies [12].

In contrast with other reports, [20, 26] age and BMI were not significant factors predicting the level of functioning after surgery in this study. However, previous studies used different time periods or different performance tests as dependent variables. Other studies [3, 8, 18] have reported findings in line with those in this study.

Our finding that mental status can have an effect on pain and functional status has also been reported by others [12, 15, 20]. However, in our study the influence of the mental status was only evident in the short term, unlike what was found in other studies [15, 20].

Several reports have described the influence of poor pre-surgical functioning on the level of functioning afterwards [1, 8, 11, 12, 21]. It was only the importance of the predictors which differed between the various studies.

In contrast to what has been reported in the literature [1], and contrary to our expectations, our study was unable to find a predictive value for the gait parameters. A reason could be our choice of the WOMAC as a dependent variable, whereas Bade et al. [1] used gait-related outcomes as dependent variables. They concluded that the pre-surgical performances on the Timed-Up-and-Go (TUG) or the Stair Climbing Test (SCT), or the distance walked in the 6-min-walk test (6MWT) predicted the post-surgical scores for the TUG, SCT and 6MWT, respectively. It seems that pre-surgical tests are mainly good at predicting their own post-surgical values.

Overall, the results of this study confirm the results of previous studies, in that pre-surgical functional status and knee status are important factors to predict the level of functioning afterwards, both in the short and longer term. Therefore, further research should investigate if improving pre-surgical factors might contribute to better outcomes in the long term.

The findings can be useful to identify patients with a poor prognosis. These patients may benefit most from the surgery, but extra attention must be paid to their physical recovery. The focus of further research could be on the effect of more, and more intensive, physical therapy on the prognosis of functional recovery.

Finally, this study has added information on the effectiveness of a TKA for the level of functioning after surgery. It could be helpful in the process of informing patients during the pre-surgical process, to give them some idea of the prognostic consequences, which is important in patient-centred care [15] and could reduce dissatisfaction after surgery. In addition, health care providers can use these results in their day-to-day clinical work to inform and benchmark their patients about their post-surgical rehabilitation, and encourage them to improve their pre-surgical functional status.

One limitation of our study is the lack of information about the specific physical therapy programmes attended by the participants, in terms of specific treatment content, attendance rates, duration, frequency and reasons for ending the therapy.

This study focused on general factors and on factors which might be influenced by physical therapy and the overall mental status, which enabled part of the variance to be explained. However, the specific influence of individual components of the mental or social status was not taken into account. Investigating a combination of these factors could improve a prediction model for the level of functioning after a TKA. Therefore, further research could focus on a combination of these factors and the pre-surgical level of functioning.

## Conclusions

Overall, TKA is a successful form of surgery, in view of the considerable overall improvement in level of functioning. The predictive value of an extensive measurement set based on all important functions and disabilities of the ICF model proved to be limited.

## Appendix

Tables 4 and 5 in appendix.

**Table 4:** Patient characteristics and baseline measurement

	*n*	Mean	95%CI
Age (years)	150	64.7	63.4–66.0
Weight (kg). Women	79	84.6	81.2–88.0
Weight (kg). Men	71	95.7	92.5–98.9
Height (m). Women	79	1.66	1.65–1.67
Height (m). Men	71	1.75	1.74–1.77
BMI. Women	79	30.7	29.5–31.9
BMI. Men	71	31.2	30.2–32.2
PSFS1	150	1.9	1.6–1.9
PSFS2	149	2.2	1.9–2.6
PSFS3	138	2.6	1.6–2.3
KSS-knee score	150	52.5	49.8–55.1
KSS-function score	150	57.2	55.1–59.4
SF12-physical	146	33.5	32.2–34.8
SF12-mental	146	44.7	42.9–46.4
Quadriceps Isometric strength 60° (Nm)	147	79.6	73.2–86.0
Quadriceps Isometric strength 30° (Nm)	132	40.1	36.3–43.8
Quadriceps Isokinetic strength 60°/s (Nm)	149	56.6	51.2–62.1
Quadriceps Isokinetic strength 180°/s (Nm)	149	35.9	32.5–39.3
Hamstrings Isometric strength 60° (Nm)	147	56.6	52.2–60.1
Hamstrings Isometric strength 30° (Nm)	132	72.2	65.9–78.4
Hamstrings Isokinetic strength 60°/s (Nm)	149	40.3	36.6–43.9
Hamstrings Isokinetic strength 180°/s (Nm)	149	30.8	28.0-33.6
Flexion	150	120.1	118.1–122.2
Extension	150	-2.3	− 3.2 to − 1.4
Walking speed (cm/s)	145	98.9	95.0–102.7
Step length surgical leg (cm)	145	57.6	55.7–59.5
Step length healthy leg (cm)	145	58.0	56.4–59.7
Stance surgical leg (%GC)	145	67.3	66.5–68.2
Stance healthy leg (%GC)	145	65.8	65.0–66.7
Swing surgical leg (%GC)	145	33.1	32.3–33.9
Swing healthy leg (%GC)	145	34.7	34.0–35.4
Single support surgical leg (%GC)	145	34.5	34.0–35.0
Single support healthy leg (%GC)	145	32.9	32.2–33.5
Double support surgical leg (%GC)	145	33.0	31.9–34.1
Double support healthy leg (%GC)	145	33.2	32.2–34.3

*n* number, *%GC* percentage of gait cycle

**Table 5:** Factors retained in multivariate regression analysis

	WOMAC3M	WOMAC12M
WOMAC-pain	0.00	0.00
WOMAC-stiffness	0.04	0.03
WOMAC-function	0.00	0.00
PSFS3	0.03	0.09
KSS-knee	ns	0.00
KSS-function	ns	0.06
SF12-physical	0.00	0.00
SF12-mental	0.01	0.09
Quadriceps isometric 60	ns	0.05
Hamstrings isokinetic 60	ns	0.01
Walking speed	ns	0.02
Step length surgical	0.07	0.00
Step length healthy	ns	0.01
Stance surgical	ns	0.08
Stance Healthy	ns	0.07
Single support surgical	0.05	0.04
Single support healthy	ns	0.04
Swing surgical	ns	0.03
Swing healthy	0.06	0.03
Double support surgical	ns	0.09
Double support healthy	ns	0.06

*r* correlation coefficient, *ns* non significant

## Abbreviations

ASA: American society of anaesthesiologists grade
BMI: Body Mass Index
KSS: Knee society score
NRS: Numeric Rating Scale
PSFS: Patient Specific Function Scale
ROM: Range of motion
SF12: Short form 12
TKA: Total knee arthroplasty
WOMAC: Western Ontario and McMaster Universities Osteoarthritis Index
WOMACF: Western Ontario and McMaster Universities Osteoarthritis Index Function Scale
